# Quantification of the aridity process in South-Western Romania

**DOI:** 10.1186/2052-336X-11-5

**Published:** 2013-05-24

**Authors:** Daniel Peptenatu, Igor Sîrodoev, Remus Pravalie

**Affiliations:** 1 Interdisciplinary Center for Advanced Research on Territorial Dynamics, University of Bucharest, 4-12 Regina Elisabeta, Bucharest, Romania; 2 Moldavian Academy of Sciences, Institute of Ecology and Geography, 1 Academiei, Chisinau MD-2028, Republic of Moldova; 3 Faculty of Geography, Bucharest University, 1, Nicolae Bălcescu str, Bucharest, Romania

**Keywords:** Aridity, Desertification, Fragile environments, Territorial management

## Abstract

The report released by the Intergovernmental Committee for Climate Change indicates that Romania ranks among the top seven countries in Europe that would be strongly impacted by aridity in the next few years, with climate changes consisting in a rise of average annual temperatures by as much as 5°C. The research work was conducted in the South of the Oltenia South-Western Development Region, where more than 700,000 hectares of farmland is impacted by aridification, more than 100,000 hectares among them impacted by aridity. Research methodology encompassed the analysis of average annual temperatures over the time span data was available for, at three weather stations, an analysis of average annual precipitations, an analysis of the piezometric data, the evolution of land use as a result of the expansion of the aridity process. The assessment of the aridity process also involved taking into consideration the state of the vegetation by means of the normalized difference vegetation index (NDVI), used to assess the quality of the vegetal stratum, an important element in the complex analysis of the territory. The aridity process is an effect of global warming, and, based on the results of this study, the post-1990 escalation of its effects was brought about by socio-economic factors. The destruction of the irrigation systems and protective forest belts because of the uncertain situation of land ownership are the main factors that contributed to amplification of the effects of aridity on the efficiency of agricultural systems that nowadays are exposed to very high risks.

## Introduction

Romania faces nowadays the obvious consequences of the aridification process on large tracts of land in the South and the East; for that reason, the United Nations Convention to combat desertification in the countries that face severe drought and/or desertification (Paris 17.06.1994) was ratified by the Law No. 111/1998. According to that document desertification and drought are acknowledged global-scale problems, and concerted actions are needed to cut down their effects at the level of territorial systems.

The analysis of the climate change has an important role due to the different implications on the agricultural systems [1], in function of the climate conditions, soil characteristics and the territorial systems capacity ability to cope with change [2-4].

The study of the desertification trend developed in many regions of the world plays an important role in the development of some territorial management strategies that are able to assure optimal functioning at the resources level, resources that have a decreasing tendency [5-8].

## Materials and methods

**Figure 1:**
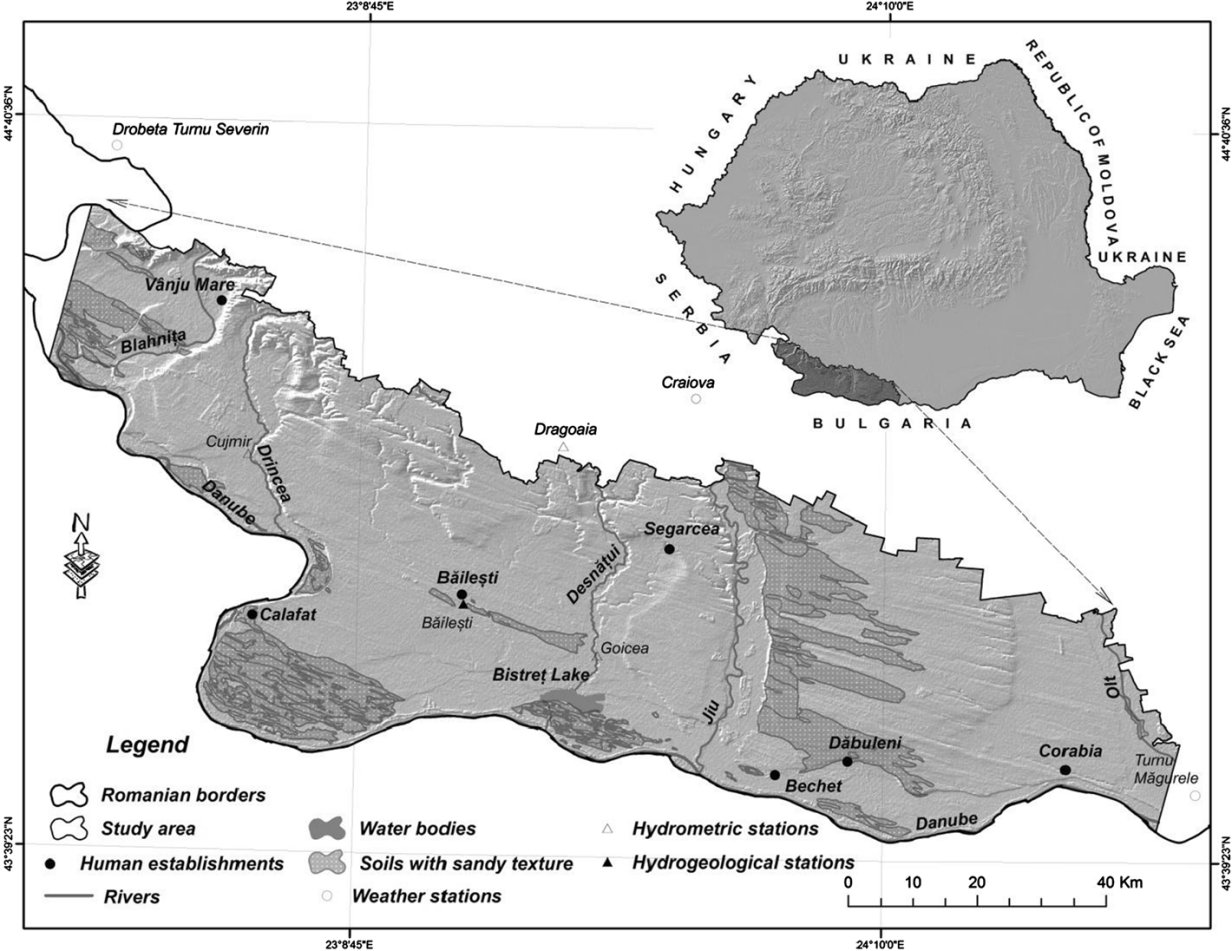
Areas risking desertification in South-Western Romania.

The assessment of the aridity process in South-Western Romania (Figure 1) was realized using historical records of climatic parameters for the period 1961-2009 (at the Craiova, Drobeta Turnu Severin and Turnu Măgurele weather stations). Detection of changes in the state of vegetation was made by the means of the normalized difference vegetation index (NDVI). NDVI was extracted through processing Landsat5TM images acquired on 07JUL1990 and 22AUG2011 [9,10].

This indicator enables an assessment of the quality of the vegetal stratum, an important component in territory planning, assessment of the state of the ecology in urbanized territories, of the evolution of land use, of the aridity process [11,12].

## Results

**Figure 2:**
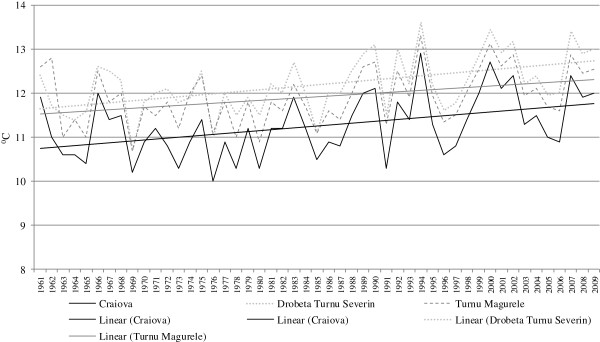
Evolution of average annual temperatures.

The analysis of the evolution of the main climate parameters at the three weather stations indicates an upward trend in average annual temperatures (Figure 2), with several differences in the type of evolution determined by local factors such as the lay of the landforms, slope gradients, the vegetation cover extent, altitude, etc.

The comparative analysis of the trends of evolution compared to the multi-annual average values indicates a series of shared characteristics for the three weather stations, the most important – for this study – being the predominance of negative deviations from the mean average until 1985 and the predominance of positive deviations until the present.

**Figure 3:**
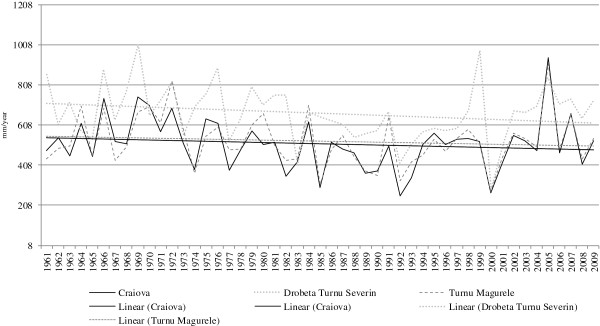
Evolution of average annual precipitations.

The analysis of the evolution of the amount of precipitations at the three weather stations indicates a general trend of decline of the amount of precipitations (Figure 3).

The comparison of the general trend and the mean average during the time span analyzed indicates a high frequency of positive-deviation intervals before 1985 and a high frequency of negative deviations after that year.

**Figure 4:**
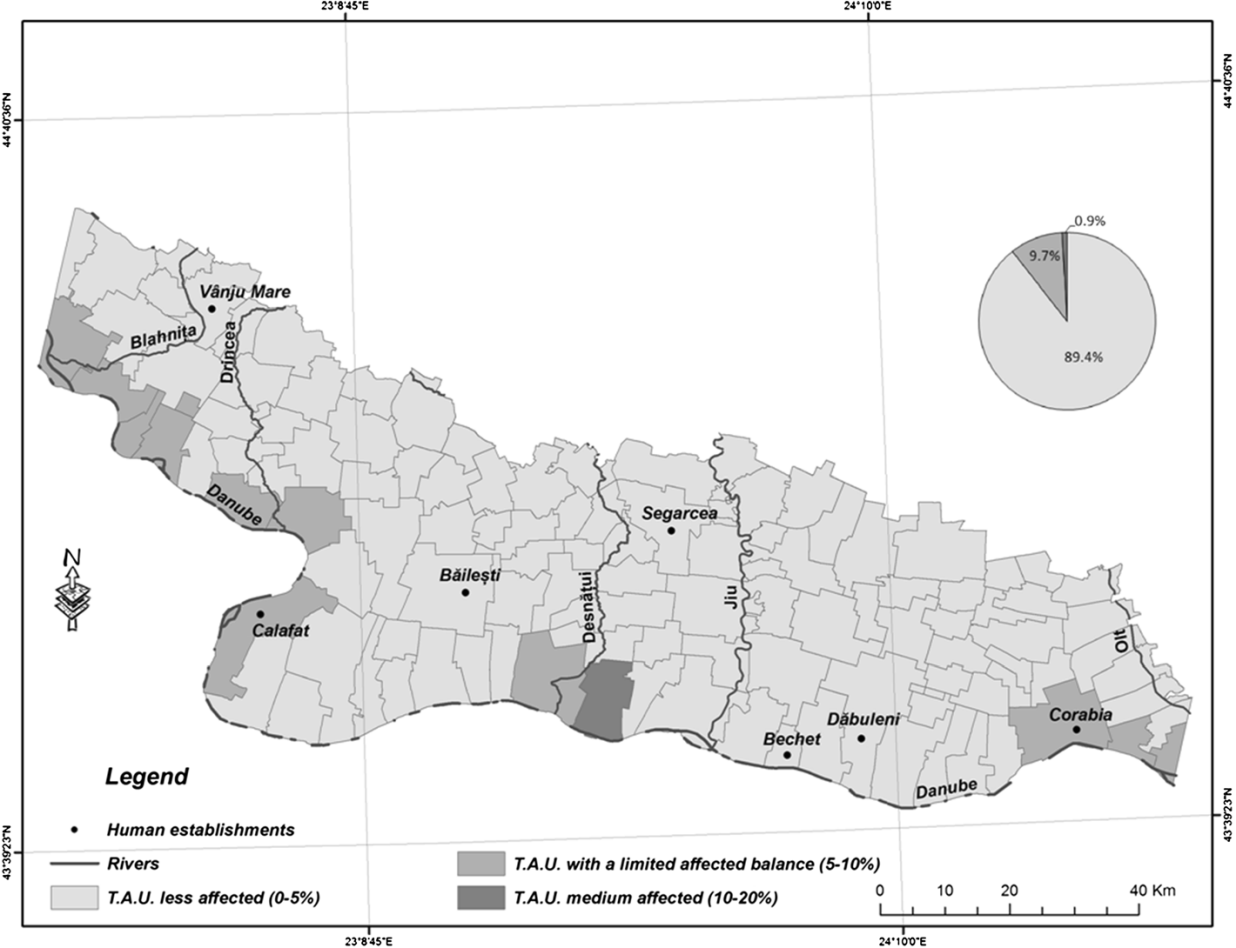
NDVI distribution-1990 (TAU-territorials administrative units).

**Figure 5:**
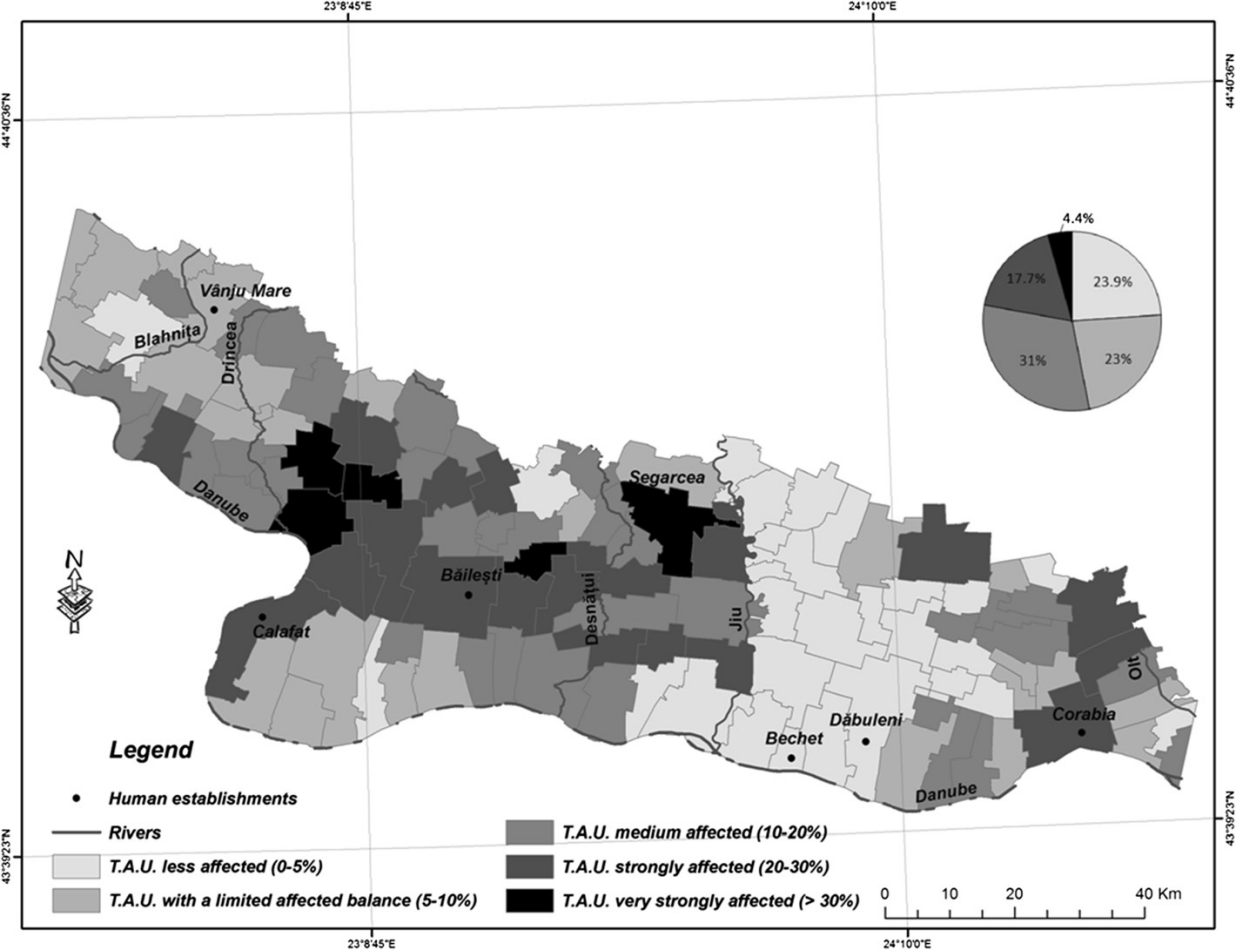
NDVI distribution-2011 (TAU-territorials administrative units).

NDVI distribution by 1990 indicates a quite well-balanced state of vegetation distribution in the area (Figure 4). More than 60% of the surface is covered by green and dense vegetation, while circa 10% is covered by very sparse vegetation or areas without any vegetation, bodies of water included. By 2011 the situation had changed radically (Figure 5). While the ratio of tracts of land covered by average-density vegetation remained more or less unchanged, the ratio of tracts of land with sparse vegetation had risen by one third, to the detriment of dense vegetation. The most spectacular changes encompassed the inner lands West of the Olt river and the lands between the Jiu river and Ohrincea.

Changes of smaller intensity, most often with transitions from the dense-vegetation category to the average-density category, but quite vast in size, were registered on the tracts of land with sandy soil in several sectors, in particular East of the Jiu and inside the Danube river bend South of Calafat. These tracts of land tend to be grouped into small, compact areas. This indicates the deterioration of the quality of the vegetal stratum in those respective areas. The soil in those areas, much more fragile because of its texture, becomes much more exposed to aridity processes.

**Figure 6:**
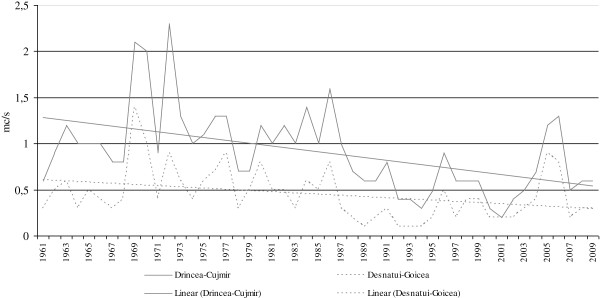
Evolution of the local river flows.

The aridity trend is revealed by the evolution of the local river flows, which in the analysed period were significantly decreased (Figure 6).

## Discussion

Aridization is a phenomenon which contributes to emphasize the fragility tendencies of the territorial systems. At a structural level, the imbalances generate functional ruptures that hinder the optimal functionality.

The agricultural production systems are the most affected by climate changes, the process of aridization generating significant reductions of agricultural production that depends more and more on climate conditions.

The diminishing of the aridisation effects requires a territorial management strategy that has to take into consideration the complexity of the impact on a short, medium and long term. Also, the decisional impulses have to consider the functional imbalances generated by aridisation at local, regional and macro-system level.

## Conclusions

Aridity is a phenomenon that contributes to increasing the fragility of the systems impacted, by multiplying the negative environmental, economic and social effects. Research into the causes that contribute to enhancing those effects is one of the priorities of contemporary society, as interdisciplinary approaches to the complex relations between the components of the territorial systems are one of the major concerns of the scientific world [13-16].

The need to implement territorial management strategies is supported by the important role played by communities in enhancing severe imbalances by wood deforestation and intensive farming on fragile soil. Under those conditions, efficient environment-risk management systems are needed, which should offer solutions to decision-making factors in the communities impacted [17-21]. In numerous regions the process of aridity led to a rise in the value of air-borne dust [22] in the wake of the destruction of forest vegetation and the draining of large tracts of wetland [23-25].

## Abbreviations

TAU: Territorials administrative units; NDVI: Normalized Difference Vegetation Index.

## Competing interests

The authors declare that they have no competing interests.

## Authors’ contributions

DP participated in the conception, or acquisition of data, or analysis and interpretation of data, participated in the given final approval of the version to be published; IS participated in the conception of the study and performed the statistical analysis; RP participated in the analysis and interpretation of data, involved in drafting the manuscript or revising. All authors read and approved the final manuscript.
